# Supplementary material for: T-CoV: a comprehensive portal of HLA-peptide interactions affected by SARS-CoV-2 mutations
Source: Nucleic Acids Res. 2021 Aug 16;50(D1):D883–7. doi: 10.1093/nar/gkab701 (PMC8385993; doi:10.1093/nar/gkab701)
Supplement: gkab701_Supplemental_File [file gkab701_supplemental_file.pdf]

T-CoV: a comprehensive portal of HLA-peptide interactions affected by SARS-CoV-2 mutations

Stepan Nersisyan[1,*], Anton Zhiyanov[1], Maxim Shkurnikov[1] and Alexander Tonevitsky[1,*]

[1] Faculty of Biology and Biotechnology, HSE University, Moscow, Russia

SUPPLEMENTARY DATA

Supplemental Table S1. SARS-CoV-2 variants used in the analysis by the date of manuscript submission (July 2021).

Variant	GISAID virus name	GISAID accession ID
Alpha 202012/01 GRY (B.1.1.7)	hCoV-19/England/MILK-9E2FE0/2020	EPI_ISL_581117
Beta GH/501Y.V2 (B.1.351)	hCoV-19/South Africa/KRISP-K004312/2020	EPI_ISL_660190
Gamma GR/501Y.V3 (P.1)	hCoV-19/Japan/IC-0561/2021	EPI_ISL_792680
Delta G/478K.V1 (B.1.617.2+AY.1+AY.2)	hCoV-19/India/ILSGS00941/2020	EPI_ISL_1663516
Epsilon GH/452R.V1 (B.1.429+B.1.427)	hCoV-19/USA/CA-CZB-12872/2020	EPI_ISL_648527
Zeta GR/484K.V2 (P.2)	hCoV-19/England/PORT-2EAFC1/2020	EPI_ISL_842052
Eta G/484K.V3 (B.1.525)	hCoV-19/England/CAMC-C769B3/2020	EPI_ISL_760883
Theta GR/1092K.V1 (P.3)	hCoV-19/Hong Kong/CM21000064/2021	EPI_ISL_1914574
Iota GH/253G.V1 (B.1.526)	hCoV-19/USA/NY-MSHSPSP-PV21166/2020	EPI_ISL_801973
Kappa G/452R.V3 (B.1.617.1)	hCoV-19/India/ILSGS00308/2020	EPI_ISL_1372093
Lambda GR/452Q.V1 (C.37)	hCoV-19/Peru/LIM-INS-869/2020	EPI_ISL_1534645

Supplemental Table S2. HLA alleles used in the study.

HLA allele	Gene	HLA class
HLA-A*01:01	HLA-A	I
HLA-A*02:01	HLA-A	I
HLA-A*02:02	HLA-A	I
HLA-A*02:06	HLA-A	I
HLA-A*02:11	HLA-A	I
HLA-A*03:01	HLA-A	I
HLA-A*03:02	HLA-A	I
HLA-A*11:01	HLA-A	I
HLA-A*23:01	HLA-A	I
HLA-A*24:02	HLA-A	I
HLA-A*25:01	HLA-A	I
HLA-A*26:01	HLA-A	I
HLA-A*29:02	HLA-A	I
HLA-A*30:01	HLA-A	I
HLA-A*30:02	HLA-A	I
HLA-A*31:01	HLA-A	I
HLA-A*32:01	HLA-A	I
HLA-A*33:03	HLA-A	I
HLA-A*68:01	HLA-A	I
HLA-A*68:02	HLA-A	I
HLA-A*74:01	HLA-A	I
HLA-B*07:02	HLA-B	I
HLA-B*08:01	HLA-B	I
HLA-B*14:02	HLA-B	I
HLA-B*15:01	HLA-B	I
HLA-B*15:03	HLA-B	I
HLA-B*18:01	HLA-B	I
HLA-B*27:05	HLA-B	I
HLA-B*35:01	HLA-B	I
HLA-B*35:03	HLA-B	I
HLA-B*38:01	HLA-B	I
HLA-B*40:01	HLA-B	I
HLA-B*40:02	HLA-B	I
HLA-B*40:06	HLA-B	I
HLA-B*42:01	HLA-B	I
HLA-B*44:02	HLA-B	I
HLA-B*44:03	HLA-B	I
HLA-B*45:01	HLA-B	I
HLA-B*50:01	HLA-B	I

HLA-B*51:01	HLA-B	I
HLA-B*52:01	HLA-B	I
HLA-B*53:01	HLA-B	I
HLA-B*57:01	HLA-B	I
HLA-B*58:01	HLA-B	I
HLA-B*58:02	HLA-B	I
HLA-C*01:02	HLA-C	I
HLA-C*02:02	HLA-C	I
HLA-C*02:10	HLA-C	I
HLA-C*03:02	HLA-C	I
HLA-C*03:03	HLA-C	I
HLA-C*03:04	HLA-C	I
HLA-C*04:01	HLA-C	I
HLA-C*05:01	HLA-C	I
HLA-C*06:02	HLA-C	I
HLA-C*07:01	HLA-C	I
HLA-C*07:02	HLA-C	I
HLA-C*08:01	HLA-C	I
HLA-C*08:02	HLA-C	I
HLA-C*12:02	HLA-C	I
HLA-C*12:03	HLA-C	I
HLA-C*14:02	HLA-C	I
HLA-C*15:02	HLA-C	I
HLA-C*16:01	HLA-C	I
HLA-C*17:01	HLA-C	I
HLA-DRB1*01:01	HLA-DRB1	II
HLA-DRB1*01:02	HLA-DRB1	II
HLA-DRB1*03:01	HLA-DRB1	II
HLA-DRB1*03:02	HLA-DRB1	II
HLA-DRB1*04:01	HLA-DRB1	II
HLA-DRB1*04:02	HLA-DRB1	II
HLA-DRB1*04:03	HLA-DRB1	II
HLA-DRB1*04:04	HLA-DRB1	II
HLA-DRB1*04:07	HLA-DRB1	II
HLA-DRB1*07:01	HLA-DRB1	II
HLA-DRB1*08:02	HLA-DRB1	II
HLA-DRB1*08:04	HLA-DRB1	II
HLA-DRB1*10:01	HLA-DRB1	II
HLA-DRB1*11:01	HLA-DRB1	II
HLA-DRB1*11:02	HLA-DRB1	II
HLA-DRB1*11:04	HLA-DRB1	II
HLA-DRB1*12:02	HLA-DRB1	II

HLA-DRB1*13:01	HLA-DRB1	II
HLA-DRB1*13:02	HLA-DRB1	II
HLA-DRB1*14:01	HLA-DRB1	II
HLA-DRB1*14:04	HLA-DRB1	II
HLA-DRB1*15:01	HLA-DRB1	II
HLA-DRB1*15:02	HLA-DRB1	II
HLA-DRB1*15:03	HLA-DRB1	II
HLA-DRB1*16:01	HLA-DRB1	II
HLA-DPA1*01:03/DPB1*01:01	HLA-DPA1/DPB1	II
HLA-DPA1*02:01/DPB1*01:01	HLA-DPA1/DPB1	II
HLA-DPA1*02:02/DPB1*01:01	HLA-DPA1/DPB1	II
HLA-DPA1*03:01/DPB1*01:01	HLA-DPA1/DPB1	II
HLA-DPA1*04:01/DPB1*01:01	HLA-DPA1/DPB1	II
HLA-DPA1*01:03/DPB1*02:01	HLA-DPA1/DPB1	II
HLA-DPA1*02:01/DPB1*02:01	HLA-DPA1/DPB1	II
HLA-DPA1*02:02/DPB1*02:01	HLA-DPA1/DPB1	II
HLA-DPA1*03:01/DPB1*02:01	HLA-DPA1/DPB1	II
HLA-DPA1*04:01/DPB1*02:01	HLA-DPA1/DPB1	II
HLA-DPA1*01:03/DPB1*03:01	HLA-DPA1/DPB1	II
HLA-DPA1*02:01/DPB1*03:01	HLA-DPA1/DPB1	II
HLA-DPA1*02:02/DPB1*03:01	HLA-DPA1/DPB1	II
HLA-DPA1*03:01/DPB1*03:01	HLA-DPA1/DPB1	II
HLA-DPA1*04:01/DPB1*03:01	HLA-DPA1/DPB1	II
HLA-DPA1*01:03/DPB1*04:01	HLA-DPA1/DPB1	II
HLA-DPA1*02:01/DPB1*04:01	HLA-DPA1/DPB1	II
HLA-DPA1*02:02/DPB1*04:01	HLA-DPA1/DPB1	II
HLA-DPA1*03:01/DPB1*04:01	HLA-DPA1/DPB1	II
HLA-DPA1*04:01/DPB1*04:01	HLA-DPA1/DPB1	II
HLA-DPA1*01:03/DPB1*04:02	HLA-DPA1/DPB1	II
HLA-DPA1*02:01/DPB1*04:02	HLA-DPA1/DPB1	II
HLA-DPA1*02:02/DPB1*04:02	HLA-DPA1/DPB1	II
HLA-DPA1*03:01/DPB1*04:02	HLA-DPA1/DPB1	II
HLA-DPA1*04:01/DPB1*04:02	HLA-DPA1/DPB1	II
HLA-DPA1*01:03/DPB1*05:01	HLA-DPA1/DPB1	II
HLA-DPA1*02:01/DPB1*05:01	HLA-DPA1/DPB1	II
HLA-DPA1*02:02/DPB1*05:01	HLA-DPA1/DPB1	II
HLA-DPA1*03:01/DPB1*05:01	HLA-DPA1/DPB1	II
HLA-DPA1*04:01/DPB1*05:01	HLA-DPA1/DPB1	II
HLA-DPA1*01:03/DPB1*06:01	HLA-DPA1/DPB1	II
HLA-DPA1*02:01/DPB1*06:01	HLA-DPA1/DPB1	II
HLA-DPA1*02:02/DPB1*06:01	HLA-DPA1/DPB1	II
HLA-DPA1*03:01/DPB1*06:01	HLA-DPA1/DPB1	II

HLA-DPA1*04:01/DPB1*06:01	HLA-DPA1/DPB1	II
HLA-DPA1*01:03/DPB1*09:01	HLA-DPA1/DPB1	II
HLA-DPA1*02:01/DPB1*09:01	HLA-DPA1/DPB1	II
HLA-DPA1*02:02/DPB1*09:01	HLA-DPA1/DPB1	II
HLA-DPA1*03:01/DPB1*09:01	HLA-DPA1/DPB1	II
HLA-DPA1*04:01/DPB1*09:01	HLA-DPA1/DPB1	II
HLA-DPA1*01:03/DPB1*10:01	HLA-DPA1/DPB1	II
HLA-DPA1*02:01/DPB1*10:01	HLA-DPA1/DPB1	II
HLA-DPA1*02:02/DPB1*10:01	HLA-DPA1/DPB1	II
HLA-DPA1*03:01/DPB1*10:01	HLA-DPA1/DPB1	II
HLA-DPA1*04:01/DPB1*10:01	HLA-DPA1/DPB1	II
HLA-DPA1*01:03/DPB1*105:01	HLA-DPA1/DPB1	II
HLA-DPA1*02:01/DPB1*105:01	HLA-DPA1/DPB1	II
HLA-DPA1*02:02/DPB1*105:01	HLA-DPA1/DPB1	II
HLA-DPA1*03:01/DPB1*105:01	HLA-DPA1/DPB1	II
HLA-DPA1*04:01/DPB1*105:01	HLA-DPA1/DPB1	II
HLA-DPA1*01:03/DPB1*11:01	HLA-DPA1/DPB1	II
HLA-DPA1*02:01/DPB1*11:01	HLA-DPA1/DPB1	II
HLA-DPA1*02:02/DPB1*11:01	HLA-DPA1/DPB1	II
HLA-DPA1*03:01/DPB1*11:01	HLA-DPA1/DPB1	II
HLA-DPA1*04:01/DPB1*11:01	HLA-DPA1/DPB1	II
HLA-DPA1*01:03/DPB1*13:01	HLA-DPA1/DPB1	II
HLA-DPA1*02:01/DPB1*13:01	HLA-DPA1/DPB1	II
HLA-DPA1*02:02/DPB1*13:01	HLA-DPA1/DPB1	II
HLA-DPA1*03:01/DPB1*13:01	HLA-DPA1/DPB1	II
HLA-DPA1*04:01/DPB1*13:01	HLA-DPA1/DPB1	II
HLA-DPA1*01:03/DPB1*14:01	HLA-DPA1/DPB1	II
HLA-DPA1*02:01/DPB1*14:01	HLA-DPA1/DPB1	II
HLA-DPA1*02:02/DPB1*14:01	HLA-DPA1/DPB1	II
HLA-DPA1*03:01/DPB1*14:01	HLA-DPA1/DPB1	II
HLA-DPA1*04:01/DPB1*14:01	HLA-DPA1/DPB1	II
HLA-DPA1*01:03/DPB1*17:01	HLA-DPA1/DPB1	II
HLA-DPA1*02:01/DPB1*17:01	HLA-DPA1/DPB1	II
HLA-DPA1*02:02/DPB1*17:01	HLA-DPA1/DPB1	II
HLA-DPA1*03:01/DPB1*17:01	HLA-DPA1/DPB1	II
HLA-DPA1*04:01/DPB1*17:01	HLA-DPA1/DPB1	II
HLA-DPA1*01:03/DPB1*26:01	HLA-DPA1/DPB1	II
HLA-DPA1*02:01/DPB1*26:01	HLA-DPA1/DPB1	II
HLA-DPA1*02:02/DPB1*26:01	HLA-DPA1/DPB1	II
HLA-DPA1*03:01/DPB1*26:01	HLA-DPA1/DPB1	II
HLA-DPA1*04:01/DPB1*26:01	HLA-DPA1/DPB1	II
HLA-DPA1*01:03/DPB1*85:01	HLA-DPA1/DPB1	II

HLA-DPA1*02:01/DPB1*85:01	HLA-DPA1/DPB1	II
HLA-DPA1*02:02/DPB1*85:01	HLA-DPA1/DPB1	II
HLA-DPA1*03:01/DPB1*85:01	HLA-DPA1/DPB1	II
HLA-DPA1*04:01/DPB1*85:01	HLA-DPA1/DPB1	II
HLA-DQA1*01:01/DQB1*02:01	HLA-DQA1/DQB1	II
HLA-DQA1*01:02/DQB1*02:01	HLA-DQA1/DQB1	II
HLA-DQA1*01:03/DQB1*02:01	HLA-DQA1/DQB1	II
HLA-DQA1*02:01/DQB1*02:01	HLA-DQA1/DQB1	II
HLA-DQA1*03:01/DQB1*02:01	HLA-DQA1/DQB1	II
HLA-DQA1*04:01/DQB1*02:01	HLA-DQA1/DQB1	II
HLA-DQA1*05:01/DQB1*02:01	HLA-DQA1/DQB1	II
HLA-DQA1*06:01/DQB1*02:01	HLA-DQA1/DQB1	II
HLA-DQA1*01:01/DQB1*02:02	HLA-DQA1/DQB1	II
HLA-DQA1*01:02/DQB1*02:02	HLA-DQA1/DQB1	II
HLA-DQA1*01:03/DQB1*02:02	HLA-DQA1/DQB1	II
HLA-DQA1*02:01/DQB1*02:02	HLA-DQA1/DQB1	II
HLA-DQA1*03:01/DQB1*02:02	HLA-DQA1/DQB1	II
HLA-DQA1*04:01/DQB1*02:02	HLA-DQA1/DQB1	II
HLA-DQA1*05:01/DQB1*02:02	HLA-DQA1/DQB1	II
HLA-DQA1*06:01/DQB1*02:02	HLA-DQA1/DQB1	II
HLA-DQA1*01:01/DQB1*03:01	HLA-DQA1/DQB1	II
HLA-DQA1*01:02/DQB1*03:01	HLA-DQA1/DQB1	II
HLA-DQA1*01:03/DQB1*03:01	HLA-DQA1/DQB1	II
HLA-DQA1*02:01/DQB1*03:01	HLA-DQA1/DQB1	II
HLA-DQA1*03:01/DQB1*03:01	HLA-DQA1/DQB1	II
HLA-DQA1*04:01/DQB1*03:01	HLA-DQA1/DQB1	II
HLA-DQA1*05:01/DQB1*03:01	HLA-DQA1/DQB1	II
HLA-DQA1*06:01/DQB1*03:01	HLA-DQA1/DQB1	II
HLA-DQA1*01:01/DQB1*03:02	HLA-DQA1/DQB1	II
HLA-DQA1*01:02/DQB1*03:02	HLA-DQA1/DQB1	II
HLA-DQA1*01:03/DQB1*03:02	HLA-DQA1/DQB1	II
HLA-DQA1*02:01/DQB1*03:02	HLA-DQA1/DQB1	II
HLA-DQA1*03:01/DQB1*03:02	HLA-DQA1/DQB1	II
HLA-DQA1*04:01/DQB1*03:02	HLA-DQA1/DQB1	II
HLA-DQA1*05:01/DQB1*03:02	HLA-DQA1/DQB1	II
HLA-DQA1*06:01/DQB1*03:02	HLA-DQA1/DQB1	II
HLA-DQA1*01:01/DQB1*03:03	HLA-DQA1/DQB1	II
HLA-DQA1*01:02/DQB1*03:03	HLA-DQA1/DQB1	II
HLA-DQA1*01:03/DQB1*03:03	HLA-DQA1/DQB1	II
HLA-DQA1*02:01/DQB1*03:03	HLA-DQA1/DQB1	II
HLA-DQA1*03:01/DQB1*03:03	HLA-DQA1/DQB1	II
HLA-DQA1*04:01/DQB1*03:03	HLA-DQA1/DQB1	II

HLA-DQA1*05:01/DQB1*03:03	HLA-DQA1/DQB1	II
HLA-DQA1*06:01/DQB1*03:03	HLA-DQA1/DQB1	II
HLA-DQA1*01:01/DQB1*03:19	HLA-DQA1/DQB1	II
HLA-DQA1*01:02/DQB1*03:19	HLA-DQA1/DQB1	II
HLA-DQA1*01:03/DQB1*03:19	HLA-DQA1/DQB1	II
HLA-DQA1*02:01/DQB1*03:19	HLA-DQA1/DQB1	II
HLA-DQA1*03:01/DQB1*03:19	HLA-DQA1/DQB1	II
HLA-DQA1*04:01/DQB1*03:19	HLA-DQA1/DQB1	II
HLA-DQA1*05:01/DQB1*03:19	HLA-DQA1/DQB1	II
HLA-DQA1*06:01/DQB1*03:19	HLA-DQA1/DQB1	II
HLA-DQA1*01:01/DQB1*04:02	HLA-DQA1/DQB1	II
HLA-DQA1*01:02/DQB1*04:02	HLA-DQA1/DQB1	II
HLA-DQA1*01:03/DQB1*04:02	HLA-DQA1/DQB1	II
HLA-DQA1*02:01/DQB1*04:02	HLA-DQA1/DQB1	II
HLA-DQA1*03:01/DQB1*04:02	HLA-DQA1/DQB1	II
HLA-DQA1*04:01/DQB1*04:02	HLA-DQA1/DQB1	II
HLA-DQA1*05:01/DQB1*04:02	HLA-DQA1/DQB1	II
HLA-DQA1*06:01/DQB1*04:02	HLA-DQA1/DQB1	II
HLA-DQA1*01:01/DQB1*05:01	HLA-DQA1/DQB1	II
HLA-DQA1*01:02/DQB1*05:01	HLA-DQA1/DQB1	II
HLA-DQA1*01:03/DQB1*05:01	HLA-DQA1/DQB1	II
HLA-DQA1*02:01/DQB1*05:01	HLA-DQA1/DQB1	II
HLA-DQA1*03:01/DQB1*05:01	HLA-DQA1/DQB1	II
HLA-DQA1*04:01/DQB1*05:01	HLA-DQA1/DQB1	II
HLA-DQA1*05:01/DQB1*05:01	HLA-DQA1/DQB1	II
HLA-DQA1*06:01/DQB1*05:01	HLA-DQA1/DQB1	II
HLA-DQA1*01:01/DQB1*05:02	HLA-DQA1/DQB1	II
HLA-DQA1*01:02/DQB1*05:02	HLA-DQA1/DQB1	II
HLA-DQA1*01:03/DQB1*05:02	HLA-DQA1/DQB1	II
HLA-DQA1*02:01/DQB1*05:02	HLA-DQA1/DQB1	II
HLA-DQA1*03:01/DQB1*05:02	HLA-DQA1/DQB1	II
HLA-DQA1*04:01/DQB1*05:02	HLA-DQA1/DQB1	II
HLA-DQA1*05:01/DQB1*05:02	HLA-DQA1/DQB1	II
HLA-DQA1*06:01/DQB1*05:02	HLA-DQA1/DQB1	II
HLA-DQA1*01:01/DQB1*05:03	HLA-DQA1/DQB1	II
HLA-DQA1*01:02/DQB1*05:03	HLA-DQA1/DQB1	II
HLA-DQA1*01:03/DQB1*05:03	HLA-DQA1/DQB1	II
HLA-DQA1*02:01/DQB1*05:03	HLA-DQA1/DQB1	II
HLA-DQA1*03:01/DQB1*05:03	HLA-DQA1/DQB1	II
HLA-DQA1*04:01/DQB1*05:03	HLA-DQA1/DQB1	II
HLA-DQA1*05:01/DQB1*05:03	HLA-DQA1/DQB1	II
HLA-DQA1*06:01/DQB1*05:03	HLA-DQA1/DQB1	II

HLA-DQA1*01:01/DQB1*06:01	HLA-DQA1/DQB1	II
HLA-DQA1*01:02/DQB1*06:01	HLA-DQA1/DQB1	II
HLA-DQA1*01:03/DQB1*06:01	HLA-DQA1/DQB1	II
HLA-DQA1*02:01/DQB1*06:01	HLA-DQA1/DQB1	II
HLA-DQA1*03:01/DQB1*06:01	HLA-DQA1/DQB1	II
HLA-DQA1*04:01/DQB1*06:01	HLA-DQA1/DQB1	II
HLA-DQA1*05:01/DQB1*06:01	HLA-DQA1/DQB1	II
HLA-DQA1*06:01/DQB1*06:01	HLA-DQA1/DQB1	II
HLA-DQA1*01:01/DQB1*06:02	HLA-DQA1/DQB1	II
HLA-DQA1*01:02/DQB1*06:02	HLA-DQA1/DQB1	II
HLA-DQA1*01:03/DQB1*06:02	HLA-DQA1/DQB1	II
HLA-DQA1*02:01/DQB1*06:02	HLA-DQA1/DQB1	II
HLA-DQA1*03:01/DQB1*06:02	HLA-DQA1/DQB1	II
HLA-DQA1*04:01/DQB1*06:02	HLA-DQA1/DQB1	II
HLA-DQA1*05:01/DQB1*06:02	HLA-DQA1/DQB1	II
HLA-DQA1*06:01/DQB1*06:02	HLA-DQA1/DQB1	II
HLA-DQA1*01:01/DQB1*06:03	HLA-DQA1/DQB1	II
HLA-DQA1*01:02/DQB1*06:03	HLA-DQA1/DQB1	II
HLA-DQA1*01:03/DQB1*06:03	HLA-DQA1/DQB1	II
HLA-DQA1*02:01/DQB1*06:03	HLA-DQA1/DQB1	II
HLA-DQA1*03:01/DQB1*06:03	HLA-DQA1/DQB1	II
HLA-DQA1*04:01/DQB1*06:03	HLA-DQA1/DQB1	II
HLA-DQA1*05:01/DQB1*06:03	HLA-DQA1/DQB1	II
HLA-DQA1*06:01/DQB1*06:03	HLA-DQA1/DQB1	II
HLA-DQA1*01:01/DQB1*06:04	HLA-DQA1/DQB1	II
HLA-DQA1*01:02/DQB1*06:04	HLA-DQA1/DQB1	II
HLA-DQA1*01:03/DQB1*06:04	HLA-DQA1/DQB1	II
HLA-DQA1*02:01/DQB1*06:04	HLA-DQA1/DQB1	II
HLA-DQA1*03:01/DQB1*06:04	HLA-DQA1/DQB1	II
HLA-DQA1*04:01/DQB1*06:04	HLA-DQA1/DQB1	II
HLA-DQA1*05:01/DQB1*06:04	HLA-DQA1/DQB1	II
HLA-DQA1*06:01/DQB1*06:04	HLA-DQA1/DQB1	II
HLA-DQA1*01:01/DQB1*06:09	HLA-DQA1/DQB1	II
HLA-DQA1*01:02/DQB1*06:09	HLA-DQA1/DQB1	II
HLA-DQA1*01:03/DQB1*06:09	HLA-DQA1/DQB1	II
HLA-DQA1*02:01/DQB1*06:09	HLA-DQA1/DQB1	II
HLA-DQA1*03:01/DQB1*06:09	HLA-DQA1/DQB1	II
HLA-DQA1*04:01/DQB1*06:09	HLA-DQA1/DQB1	II
HLA-DQA1*05:01/DQB1*06:09	HLA-DQA1/DQB1	II
HLA-DQA1*06:01/DQB1*06:09	HLA-DQA1/DQB1	II
